# Why the uptake of eRehabilitation programs in stroke care is so difficult—a focus group study in the Netherlands

**DOI:** 10.1186/s13012-018-0827-5

**Published:** 2018-10-29

**Authors:** B. Brouns, J. J. L. Meesters, M. M. Wentink, A. J. de Kloet, H. J. Arwert, T. P. M. Vliet Vlieland, L. W. Boyce, L. van Bodegom-Vos

**Affiliations:** 1 Faculty of Health, Nutrition and Sports, The Hague University for Applied Sciences, The Hague, The Netherlands; 2 Department of Biomedical Data Sciences, Section Medical Decision Making, Leiden University Medical Center, Leiden, The Netherlands; 3 Sophia Rehabilitation Centre, The Hague, The Netherlands; 4 Rijnlands Rehabilitation Centre, Leiden, The Netherlands; 5 Department of Orthopaedics, Rehabilitation Medicine and Physical Therapy, Leiden University Medical Center, J11 Albinusdreef 2, 2333 ZA Leiden, The Netherlands

**Keywords:** Stroke, Barriers and facilitators, Implementation, Rehabilitation, eHealth, Focus groups, eRehabilitation

## Abstract

**Background:**

The uptake of eRehabilitation programs in stroke care is insufficient, despite the growing availability. The aim of this study was to explore which factors influence the uptake of eRehabilitation in stroke rehabilitation, among stroke patients, informal caregivers, and healthcare professionals.

**Methods:**

A qualitative focus group study with eight focus groups (6–8 participants per group) was conducted: six with stroke patients/informal caregivers and two with healthcare professionals involved in stroke rehabilitation (rehabilitation physicians, physical therapists, occupational therapists, psychologists, managers). Focus group interviews were audiotaped, transcribed in full, and analyzed by direct content analysis using the implementation model of Grol.

**Results:**

Thirty-two patients, 15 informal caregivers, and 13 healthcare professionals were included. A total of 14 influencing factors were found, grouped to 5 of the 6 levels of the implementation model of Grol (Innovation, Organizational context, Individual patient, Individual professional, and Economic and political context). Most quotes of patients, informal caregivers, and healthcare professionals were classified to factors at the level of the Innovation (e.g., content, attractiveness, and feasibility of eRehabilitation programs). In addition, for patients, relatively many quotes were classified to factors at the level of the individual patient (e.g., patients characteristics as fatigue and the inability to understand ICT-devices), and for healthcare professionals at the level of the organizational context (e.g., having sufficient time and the fit with existing processes of care).

**Conclusion:**

Although there was a considerable overlap in reported factors between patients/informal caregivers and healthcare professionals when it concerns eRehabilitation as innovation, its seems that patients/informal caregivers give more emphasis to factors related to the individual patient, whereas healthcare professionals emphasize the importance of factors related to the organizational context. This difference should be considered when developing an implementation strategy for patients and healthcare professionals separately.

## Background

Stroke is a major cause of disability [1], including long-term problems with motor function, cognition, communication [2], and participation [3]. Specialized rehabilitation has shown to be effective in recovery of these functions [4]. Due to the increasing incidence of stroke [5], an increased need for rehabilitation care is expected in the future [6]. To meet this increasing demand and at the same time limit the growth of stroke rehabilitation costs, blended care in which information and communication technology (ICT) are used alongside conventional therapy offers a potential solution. ICT is in the last decennia increasingly accessible, affordable, and remotely available 24/7. ICT can for example be used to relieve healthcare professionals from manual labor and make rehabilitation accessible to a larger number of stroke patients while maintaining or increasing the effectiveness of rehabilitation [7].

The use of ICT in rehabilitation, eRehabilitation, can be seen as an additional way of delivering stroke rehabilitation, in which service is delivered via a wide variety of possible ICT-devices like telephone, computer, tablets, smartphone, virtual reality, and robotic devices. It can target communication, cognitive problems, motor deficits or aphasia [8], and includes physical and cognitive exercise programs, serious gaming, goal setting, education, information [8, 9], and e-consultations for remote communication and monitoring [10]. Recent randomized clinical trials have shown that eRehabilitation programs are effective in improving health of stroke survivors [11, 12]. In addition, eRehabilitation may facilitate self-directed home-based rehabilitation, decrease chronic disability after, e.g., stroke, cardiac arrest, COPD [13], and offers possibilities to continue treatment after discharge from rehabilitation [9].

Literature about the perspective and acceptance of technologies like eRehabilitation in both patients [14–16] and healthcare professionals [17, 18] showed that most stakeholders are interested in eRehabilitation after stroke; among others to improve communication, including the possibility to call healthcare professionals in case of questions or concerns and improve social contact between patients, to increase participation in therapeutic activities and adherence, and to facilitate better rehabilitation outcomes.

Despite this positive view of the end-users and widespread agreement about the importance and potentials of eRehabilitation, use of eRehabilitation in clinical practice is lacking [19]. Literature of the last decade shows that acceptance and willingness to adopt eRehabilitation in stroke rehabilitation is hampered by the fact that not all patients are confident with ICT-devices like smartphone and tablet [14] and both patients [15, 16] and healthcare professionals [18] do not want eRehabilitation to replace more social face-to-face contact. A study about the uptake of eRehabilitation in India concluded that healthcare professionals were especially worried about adapting the existing workflow into a new way of service delivery [20]. Concerns about installation of and using ICT-devices, the lack of face-to-face contact, the limited scope of exercise, and stroke-related impairments were raised as well [20]. When using tablet-based therapies, patient had the most difficulties with following complex instructions when trying to understand how to use ICT-devices [8]. Besides, as requirement for successful uptake, healthcare professionals have stressed the importance of tailoring a program to the patients’ personal situation [18] and having sufficient time for the uptake of such innovations [17].

Although abovementioned studies give some insight into the possibilities and feasibility of eRehabilitation and characteristics of its end-users, a thorough investigation of all barriers and facilitators for the uptake of eRehabilitation for stroke in a western country, including opinions of multiple end-users, is lacking. To improve this uptake and support healthcare professionals and patients in the use of eRehabilitation, such insights are needed [14, 21]. Therefore, this study aimed to identify factors influencing in the uptake of eRehabilitation after stroke among patients, informal caregivers, and healthcare professionals.

## Methods

### Design

To identify factors influencing the uptake of eRehabilitation, a qualitative focus group study was conducted among stroke patients, informal caregivers, and healthcare professionals. Focus groups were chosen as method since this type of data collection contributes to a better understanding of end-users’ attitudes, experiences, and expectations [22]. In order to allow participants to speak freely about their treatment and experiences in the rehabilitation center, separate groups were organized for patients/informal caregivers and healthcare professionals. Moreover, it was expected that separate groups would stimulate more discussion since participants have shared experiences. The intended group size was six to eight participants, but up to ten patients were invited to account for participants who declined at short notice [23]. We planned to continue with focus group interviews until data saturation was reached. Data saturation was reached when no additional factors emerged during three consecutive interviews [24]. The COREQ guidelines were used for adequate design and reporting of the study [25].

### Recruitment and inclusion

Patients, informal caregivers, and healthcare professionals were recruited from two Dutch rehabilitation centers; Sophia Rehabilitation in The Hague and Rijnlands Rehabilitation Centre in Leiden.

#### Patients/informal caregivers

In January 2016, the electronic patient registries of the rehabilitation centers were searched for potentially eligible patients based on the following inclusion criteria: (1) older than 18 years, (2) diagnosed with stroke, and (3) completed rehabilitation which started after June 2011. From a group of approximately 2700 potential participants which are treated in 1 of both rehabilitation centers, 200 patients from each center were randomly selected. Those 400 patients received a letter with information about the study and an invitation to participate. Invitations to patients were directed to the informal caregiver as well, which could be a partner, child, parent, or friend who is involved in the daily life of the patient. An invitation was also sent to five former stroke patients who met on a regular basis to discuss on-going innovation and research projects in rehabilitation (“innovation partners”).

The invitation included a self-developed questionnaire concerning impairments as a consequence of stroke (physical, communication, cognition), use of ICT-devices (smartphone, tablet, laptop, pc), and the purpose of this use (applications, email, information, games, exercises). This was done to select a diverse group of patients with respect to type of impairments and the use of ICT-devices within each focus group.

Patients and informal caregivers could indicate their willingness to participate by filling in the informed consent and their availability for the focus groups. Patients willing to participate were selected to be part of the focus groups based on their availability and type of impairment. Some patients were not invited because of their availability. Use of ICT-devices was comparable for all participants. No reminders were sent since the number of patient that responded without reminder was expected to be high enough to reach data saturation. All responding patients and informal caregivers received an e-mail informing them whether they were invited for a focus group or not.

#### Healthcare professionals

Certified healthcare professionals (rehabilitation physician, physical therapist, occupational therapist, psychologist, and speech therapist) with at least 2 years of work experience in a specialized rehabilitation team for stroke patients were invited for the focus groups (*n* = 56, 29 at Sophia Rehabilitation, 27 at Rijnlands Rehabilitation Centre). All eligible healthcare professionals received an email with information about the study and an invitation to participate.

### Focus group

Each focus group was conducted by three persons; a moderator (MW; MSc, female/BB; MSc, female), an assistant (BB; MSc, female/HB; MSc, female), and an observer (HB; MSc, female/SH; physiotherapist, male/PK; MD, female). The assistant contributed with questions, made sure all participants were involved in the discussion, and managed the tape-recorders and time, the observer observed, and took notes. The moderator and assistant have a master’s degree in health sciences or human movement sciences and were involved in research projects in the rehabilitation center but not in daily care practice. Their education included training in the conduct of interviews and both were not involved in care of the participants. The participants had no personal background information on the interviewers.

The focus groups lasted 2 h, including a 15-min break. More breaks were provided if necessary. At the end, all patients received travel cost reimbursement and were rewarded with a gift card of €10, for participating in the focus group. Patients received feedback of the results of the focus groups by means of a newsletter. The focus groups took place between January and March 2016 in the two involved rehabilitation centers in the Netherlands.

### Interview guideline

A semi-structured interview guide was developed, including open-ended questions in the following domains: (1) the content of an eRehabilitation service, (2) appearance and accessibility, and (3) factors influencing the uptake.

Multiple models are designed to describe and categorize factors that influence the uptake of an innovation in healthcare. Example are the implementation model by Grol [26], the model by Cabana [27], determinants of change model by Fleuren [28], or consolidated framework by Damschroder [29]. For this study, the implementation model of Grol was chosen since it offers a framework to identify and categorize factors in the uptake of innovations in healthcare [26]. Especially, the innovation, in this case eRehabilitation, is included in the model, which is expected to be of major influence on the implementation.

The model of Grol suggests that the following groups of factors can be defined: (1) Innovation; in this case eRehabilitation, including advantages of its use in practice and the feasibility, accessibility, and attractiveness of eRehabilitation programs; (2) Organizational context; for example organization of care practices, staff, capacities, resources, structures; (3) Individual patient; for example knowledge, skills, and attitude of the patients, including stroke-specific characteristics; (4) Individual professionals; for example, the awareness, knowledge, skill, and motivation to change of the healthcare professionals working in the rehabilitation center; (5) Economic and political context; including financial arrangements, regulations, and policies; and (6) Social context; including opinion of colleagues, culture of the network, and collaboration.

Each focus group started with an introduction, including the aim of the meeting, timeline, and rules. Participants also gave permission for audio recording. During this introduction, a global idea about eRehabilitation was given, in which it was explained what eRehabilitation is and an example was shown on a screen. Prompts (e.g., example of eRehabilitation, pictures, questions, etc.) were included in the interview guide to support participants in verbalizing thoughts about an abstract concept as eRehabilitation. Examples of questions asked are: “What do you need in order to be able to use eRehabilitation in daily practice?” or “What kind of problems do you anticipate when using eRehabilitation?”

A pilot focus group with the five innovation partners was conducted to test the interview guide. Although they did not meet the inclusion criterion of start rehabilitation after June 2011, the pilot session did not lead to major changes in the study protocol. The data collected were added to the study data.

### Data analysis

The audio-tapes of all focus groups were transcribed in full. The transcripts were then qualitatively analyzed by two of four researchers separately [MW/BB/PK/SH]. Directed content analysis was used, in which the researchers used a theory or relevant research findings as guidance for initial coding [30], in this case the implementation model of Grol [26]. During these analyses, transcripts were read and quotes were marked with a code. Discrepancies between researchers were discussed in order to reach consensus. If researchers still disagreed, a third researcher (JM) who was not involved in the analysis made a final decision. All quotes with a code were collected in one database. Codes with comparable content were merged into sub-factors, sub-factors with comparable content were merged into factors, which were then assigned to the overarching levels of the model of Grol. Additionally, the (sub-) factors identified were discussed by the research group. Transcripts were not returned to the participants for correction. The software package Excel 2010 was used to organize codes, (sub-) factors, and levels. Descriptive statistics are used to describe basic characteristics of patients and informal caregivers.

### Ethical approval

All participants gave written informed consent prior to participation. Patients were assured their anonymity and told that participation in the study would not affect their treatment position in the rehabilitation center. The study was approved by the Medical Ethical Review Board of the Leiden University Medical Center [P15.281].

## Results

### Participants

#### Patients/informal caregivers

**Fig. 1:**
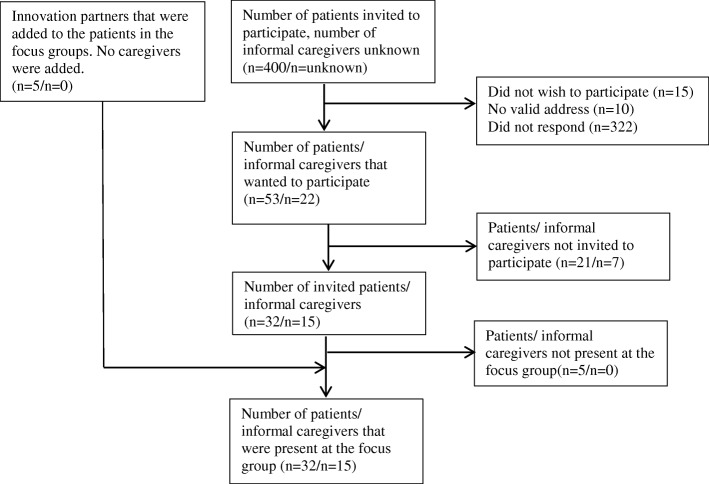
Flow of inclusion of participants in the focus group study

A total of 53 patients (response rate 13.3%) and 22 informal caregivers responded to the invitation (see Fig. 1). Six focus groups (including the pilot session with the five innovation partners) were conducted with a total number of 32 patients and 15 informal caregivers; 26 patients and 7 informal caregivers were not available at the scheduled time of the focus groups. Basic characteristics of patients and informal caregivers are shown in Table 1.

**Table 1:** Characteristics of participating stroke patients, informal caregivers, and healthcare professionals

Characteristic	Patients (*n* = 32)	Informal caregivers (*n* = 15)	Healthcare professionals (*n* = 13)
Sex; number male (%)	19 (59)	4 (27)	3 (23)
Patient and informal caregiver			
Mean age in years (SD)	56.9 (15.1)	60.6 (9.9)	
Time since stroke in months (SD)	27.8 (14.0)		
Communication problems; number (%)	16 (50)		
Motor problems; number (%)	20 (63)		
Cognitive problems; number (%)	24 (75)		
Using digital devices in daily life; number (%)	32 (100)		
Purpose of using digital devices:			
Email; number (%)	18 (56)		
Applications; number (%)	15 (47)		
Searching information; number (%)	10 (31)		
Games; number (%)	14 (44)		
Exercises; number (%)	8 (25)		
Profession			
Physiotherapist; number (%)			3 (23)
Psychologist; number (%)			1 (8)
Occupational therapist; number (%)			3 (3)
Speech therapist; number (%)			1 (8)
Rehabilitation specialist; number (%)			4 (31)
Manager; number (%)			1 (8)

*SD* standard deviation

#### Healthcare professionals

In total, 24 of the 56 healthcare professionals agreed to participate in the study (response rate 43%). Eleven healthcare professionals were not able to be present at the scheduled time, so 13 healthcare professionals were included, divided in 2 focus groups. These healthcare professional groups included physiotherapists, psychologists, occupational therapists, speech therapists, physicians, and managers (see Table 1).

### Factors influencing the uptake of eRehabilitation

From the transcripts of the 8 focus groups, quotes from patients, informal caregivers, and healthcare professionals could be merged into 21 sub-factors, which could subsequently be merged into 14 factors (see Table 2). These factors were allocated to five out of the six levels of the implementation model of Grol; Innovation, Organizational context, Individual patient, Individual professional, and Economical and political context. No factors were identified at the level of the Social context.

**Table 2:** Factors influencing the uptake of eRehabilitation programs after stroke

Level	Factor	Sub-factor	Patient and informal caregiver	Professional
Innovation	Accessibility	Period in which eRehabilitation is accessible	x	x
		Devices on which eRehabilitation is accessible	x	x
	Feasibility	Helpdesk function	x	x
		Tailored to patients’ situation	x	x
	Attractiveness	Ease of use of eRehabilitation	x	x
		Content of eRehabilitation program	x	x
	Privacy	Privacy and safety of patient data	x	x
	Advantages of use	Characteristic of innovation offering added value	x	x
Organizational context	Organization of care	Tasks and responsibilities healthcare professional	x	x
		Tasks and responsibilities informal caregiver	x	x
		Tasks and responsibilities organization		x
	Resources	Software	x	x
		Hardware		x
		Space at home	x	
	Time	Time	x	x
Individual patients	Motivation to change	Reasons (not) to use eRehabilitation for patients	x	x
	Knowledge	Knowledge about use of eRehabilitation	x	
	Skill	Skills with use eRehabilitation	x	
	Patient characteristics	Impairment after stroke	x	x
Individual professional	Motivation to change	Reasons (not) to use eRehabilitation for professionals		x
Economic and political context	Financial arrangements	Insurance	x	x

In the transcripts of the focus groups with patients/informal caregivers, 18 sub-factors could be identified. Most quotes of patients and informal caregivers were at the level of the Innovation (*n* = 234, 42% of total number of quotes) and the level of the Individual patient (*n* = 226, 40% of total number of quotes). From the transcripts of the focus groups with healthcare professionals, also 18 sub-factors could be identified. Most quotes of healthcare professionals were at the level of the Innovation (*n* = 108, 39% of total number of quotes), and the level of the Organizational context (*n* = 89, 35% of total number of quotes).

For the patients/informal caregivers, no new factors emerged after three focus groups; for the healthcare professionals, both focus groups resulted in new factors. In the following sections, the factors within each level will be discussed, first for the patient/informal caregiver and then for the healthcare professional.

#### Factors at the level of innovation (eRehabilitation program)

This level included the following factors: accessibility, feasibility, attractiveness, privacy, and advantage of use.

##### Accessibility

Patient and informal caregivers reported that the uptake of eRehabilitation would be limited when the accessibility of the eRehabilitation programs was restricted to the rehabilitation center or to their clinical rehabilitation time. “I think it should be a continuation of regular rehabilitation” (Informal caregiver 2.1). Furthermore, an eRehabilitation program should be accessible on multiple devices like a computer, laptop, tablet or smartphone.

Healthcare professionals agreed that eRehabilitation should be accessible for the patient during and after rehabilitation, on a device that patients preferred.

##### Feasibility

Patients/informal caregivers felt that eRehabilitation was only feasible when (1) a helpdesk for assistance in case of problems with the uptake of eRehabilitation programs was available, (2) the eRehabilitation program would be tailored to the patients’ personal situation, and (3) eRehabilitation would be supplemental to conventional therapy. Patients said eRehabilitation cannot replace traditional rehabilitation because patients felt they needed daily care at the start of rehabilitation and severe motor problems cannot be solved by a digital program. “At the start I could not speak or swallow. How does a program [eRehabilitation] teach me that?” (Patient 1.5).

Healthcare professionals reported the importance of an ICT-helpdesk to address technical questions about, e.g., internet connection*.* “I don’t want that we as therapists get all those questions about the program and installation of it. Where should patients go with their questions? I think a helpdesk.” (Healthcare professional 2.4). Additionally, they agreed that eRehabilitation cannot replace traditional rehabilitation and mentioned that patients also benefit from peer contact in the rehabilitation center.

##### Attractiveness

Attractiveness of an eRehabilitation program was influenced by its ease of use and content. Patients/informal caregivers reported that an eRehabilitation program should, among others, consist of cognitive and physical exercises, serious games, information, peer contact, goal setting, an agenda, and an exercise schedule. Ease of use would increase when all components of an eRehabilitation program are organized on one website, icons are used instead of text and no noise, flash signals or unclear layout was used and the design should be adjustable to personal preferences*.* “Maybe with a sweet voice, or a sweet little music.” (Informal caregiver 3.1).

Healthcare professionals mentioned that an eRehabilitation program would benefit from the inclusion of a clear day schedule with planned and performed exercises. The uptake of an eRehabilitation program would decrease if not all exercises healthcare professionals want to prescribe are included, and ease of use would decrease if it would not be possible to set up an exercise program easily.

##### Privacy

Patients did not perceive it as a violation of their privacy when a therapist would have access to their personal data. Even more, patients reported that it would be extra motivating when healthcare professionals were able to see the exercises they did (not) perform*:* “For me, it [access for the healthcare professional to exercises data] would be very motivating, since your performance is monitored.” (Patient 2.2).

For healthcare professionals, (internet) connections which could not guarantee the privacy and safety of personal data was a barrier in the uptake of eRehabilitation. “It is a must that eRehabilitation programs meets the privacy requirements. Data transport must be safe.” (Healthcare professional 1.1).

##### Advantage of use

Patients reported many advantages of eRehabilitation. Among others, this included the possibility to have a clear overview of planned and performed exercises and perform those exercises at a time of their own preference. Furthermore, it offers a possibility to continue an exercise program after discharge. A patient described this as “not feeling abandoned after discharge from the rehabilitation center.” (Patient 2.4). Besides, patients reported a possible benefit from receiving feedback about daily activities and performed exercise.

These advantages of use were also reported by healthcare professionals. “The advantage for patients is the possibility to continue exercising, which is not limited to the rehabilitation center anymore.” (Healthcare professional 2.1). In addition, the professionals also reported the possibility to have an e-consult with patients as an advantage.

#### Factors at the level of organizational context

At the level of the Organizational context, three factors were identified: organization of care, resources, and time.

##### Organization of care

Patients reported that healthcare professionals needed to set up and adjust an eRehabilitation exercise program, since patients perceived they were unable to do this themselves. “They [the healthcare professionals] obviously know the patient. So, I mean, they can say, this is what the patient needs, and adjust the program after a certain time” (Patient 4.1). The presence of an informal caregiver who could assists the patient was reported as a beneficial bonus that could increase the uptake of eRehabilitation. “She has plenty of time to learn how to use eRehabilitation but she needs someone to practice it with.” (Informal caregiver 4.6).

In line with the patients, healthcare professionals reported that an exercise program needed to be tailored to the patient’s situation, and set up by a healthcare professional. This was supplemented with the task of the organization to ensure a good fit with the existing processes of care, and to arrange all necessary software and hardware. “I think that just all the computers in the rehabilitation center must be sufficiently updated with all necessary software.” (Healthcare professionals 2.6).

##### Resources

Resources needed for successful uptake included software, hardware, and physical space. Problems with the software were reported as limiting for the uptake of eRehabilitation in all focus groups sessions. Patients/informal caregivers said they would not use eRehabilitation when problems with the software occurred which were not resolved quickly. Concerning the hardware, patients were willing to purchase required hardware like a tablet when necessary. Besides, some patients reported not having enough space (3 × 3 m) at home to perform exercise safely.

For healthcare professionals, problems with the software were mentioned as a major barrier as well. “When you plan an e-consultation with the patient and the internet connection is bad or the webcam fails, you have to reschedule the consultation. I see some large potential problems.” (Healthcare professional 2.2). Additionally, some healthcare professionals expected that the uptake among patients would be less when it was required to buy a new device, while others mentioned that most patient possess one or more ICT-devices.

##### Time

Some patients reported that the uptake of eRehabilitation would be limited due to lack of time, others perceived an eRehabilitation programs as useful daytime activity. “I was sick and had no work, so the use of eRehabilitation would have been a welcome change” (Patient 3.1).

Healthcare professionals reported that the uptake of eRehabilitation would decrease if they lacked the time to get to know the program, for instance by education from the supplier. “A reason why I do not use eRehabilitation, is because I am not familiar with all the possibilities. It takes time to make it my own, leaving less time for the patients.” (Healthcare professional 1.5). Besides, lack of time to monitor the progress of patient in the eRehabilitation program was reported as barrier as well.

#### Factors at the level of individual patient

Quotes at this level could be grouped into the factors motivation to change, skills, knowledge, and patient characteristics.

##### Motivation to change

A motivation to start using eRehabilitation was, among others, the possibility to have peer contact with other stroke patients or other informal caregivers. In addition, patients frequently mentioned that exercises would be more stimulating using eRehabilitation, since a variety of games or exercises would be more fun than exercises on paper. Reasons not to use eRehabilitation were the chance of getting overstimulated by using ICT-devices, and the replacement of personal contact by digital contact. Contact via an eRehabilitation program was perceived less personal than face-to-face contact. “You cannot replace human contact with contact by digital devices. That is always a loss.” (Patient 2.5).

Healthcare professionals reported that eRehabilitation would be motivating for patients since it would give them the opportunity to exercise outside treatment hours and after discharge or could reduce travel time and costs if e-consultations were available. However, healthcare professionals were, like patients, also afraid for overstimulation of the patients and loss of social contact. “What I hear from many clients, especially on the long-term, is loneliness. There are possibilities to prevent loneliness, but I think this [eRehabilitation] is an individualistic way of training.” (Healthcare professional 1.1).

##### Skills and knowledge

Opinions about skills and knowledge to use ICT-devices for eRehabilitation programs differed within patients and within informal caregivers. Some patients and informal caregivers reported that their skills and knowledge would be sufficient: “I can deal well with a smartphone, a tablet or a laptop.” (Patient 5.2). Other patients and informal caregivers reported not having enough knowledge or skills for the uptake of eRehabilitation programs, “I am also alone and I am not very technical, so it cannot do it on my one.” (Patient 2.2). Patients reported that they need to be taught how to use the eRehabilitation program by a healthcare professional.

Healthcare professionals did not report any factors related to skills and knowledge at the level of the individual patient.

##### Patient characteristics

Patients and informal caregivers agreed that the use of an eRehabilitation program would not be suitable for every stroke patient, due to varying impairments and limitations. Among others, these limitations could concern the loss of the ability to understand ICT-devices or loss of energy due to their stroke. Several informal caregivers mentioned that eRehabilitation was not suitable for their partner or family member. “It [handling ICT-devices] does not work now. Every time you join the group class it goes well, but when you come home you do not know how to do it anymore.” (Informal caregiver 6.3 talking to partner).

Healthcare professionals also mentioned that eRehabilitation would not be feasible for all patients in rehabilitation, but others reported that they are willing to try. “Sometimes, I want try it with a patient but I do not know if it is feasible. Then the patient really likes it and you can see another side of him; the person is very fanatical and is being active, that is very surprising.” (Healthcare professional 1.5).

#### Factors at the level of individual professional

Only from the transcripts of the healthcare professionals, one factor assigned to the level of the individual professional was identified: motivation to change, in other words why a healthcare professional would or would not start using eRehabilitation programs. Healthcare professionals expected that working as a multidisciplinary team would be easier after the uptake of an eRehabilitation program. An eRehabilitation program could improve insight in the prescribed excises and actions taken by other disciplines. Healthcare professionals mentioned that they were cautious to prescribe eRehabilitation for a longer time since they were afraid to give false hope if it was advertised that eRehabilitation program would be accessible forever. Healthcare professional: “A forever-accessible program could imply that exercising via an eRehabilitation program would be useful in the chronic phase after stroke, while most exercises promote improvement only in the period directly after stroke.” (Healthcare professional 2.1).

#### Factors at the level of economic and political context

Financial arrangements, in particular reimbursement, were the only factor identified at this level.

Some patients said that the absence of reimbursement of the costs of an eRehabilitation program made it impossible for them to start using eRehabilitation, since they could not spare the money to pay for it. Others perceived it as an extra motivation to actually use eRehabilitation when paid for it. “If it is for free, you working less hard for that.” (Informal caregiver 3.1). “So a certain payment seems good to me.” (Patient 3.2) “Or a subscription.” (Informal caregiver 3.1). “Yes, that would reinforces the involvement.” (Patient 3.2).

Healthcare professionals mentioned the absence of reimbursement only as a restricting factor for the uptake of eRehabilitation. “Implementation of eRehabilitation costs a reasonable amount of money. There is no direct return of the investment or reimbursed yet. So that is still a big bottleneck.” (Healthcare professional 1.2).

## Discussion

This qualitative focus group study explored factors influencing the uptake of eRehabilitation programs in stroke care in western country, from the perspective of patients, informal caregivers, and healthcare professionals. Fourteen factors influencing the uptake were identified, grouped into 5 levels: Innovation, Organizational context, Individual patient, Individual professional, and the Economic and political context. No factors related to the social context were found.

Considerable overlap between patients/informal caregivers and healthcare professionals was found, especially at the level of the Innovation. Many participants expressed positive beliefs about the potentials of eRehabilitation, like the possibility to continue therapy after discharge and more motivation for therapy-related activities. However, all end-users emphasized the importance of the possibility to get to know the eRehabilitation program; for patients, this included education from their healthcare professionals how to use the program; for the healthcare professionals, this included education and time to get used to the program. Differences between patients/informal caregivers and healthcare professionals were found as well. Patients/informal caregivers reported more quotes in the level of the Individual patient (i.e., patients’ characteristics as fatigue and the inability to understand ICT-devices), where healthcare professionals reported more at the level of the Organization context (i.e., having sufficient time and the fit with existing processes of care). Therefore, end-users were focused in the same extent to factors related to the Innovation, but patients/informal caregivers were more concerned about factors related to the Individual patients where healthcare professionals were more concerned about factors related to the Organizational context.

Concerns about the Organizational context were found before in the implementation of eRehabilitation in stroke [20]. Although it seems clear that eRehabilitation will affect the way daily rehabilitation is delivered [10], previous research stated that rehabilitation therapy should start with face-to-face contact to establish a good patient-professional relationship [31]. The current research stresses the importance of supplementing eRehabilitation to traditional rehabilitation instead of replacing it as well; all end-users reported that eRehabilitation would only be feasible when added to traditional rehabilitation. Therefore, to optimize stroke rehabilitation, it seems best to offer blended care in which eRehabilitation programs are added to regular face-to-face treatment and to integrate care supported by ICT with traditional care [14, 32].

This study did not find any factors related to the social context. This is in line with findings from previous studies that assessed factors influencing the implementation of eRehabilitation [10, 33, 34]. A previous study about the implementation of virtual reality [34] reported, for example, only factors related to the organizational context, individual patient, healthcare professional, and technological aspects. In addition, a policy statement reported as well, only legal, technological, and financial barriers [10]. Also after implementation, during the actual use of eRehabilitation, healthcare professionals were not worried about social pressures of colleagues [33]. A possible explanation is that the use of eRehabilitation in our study would be voluntary. The study of Schaper and Pervan [35] showed that voluntary use of technologies in the rehabilitation setting, healthcare professionals’, especially physical and occupational therapists’, intention to use eRehabilitation were not significantly influenced by colleagues; the decisions to use eRehabilitation was made independent from other team members. As a result, factors related to the social context had little influence on healthcare professionals’ uptake of technologies like eRehabilitation.

Next to the results considering blended care and the absence of factors in the social level, the results of our study confirm findings from previous studies. Healthcare professionals previously stressed the importance of getting the time and opportunity to become familiar with eRehabilitation programs [17]. In addition and in line with our findings, support of informal caregivers and the role of the healthcare professional to introduce eRehabilitation to the patients seemed crucial for successful uptake [31]. Important aspect for the feasibly of eRehabilitation is the usability for those with less capabilities and adjusted to characteristics of those clinical conditions [10].

Additional to the observed similarities, differences between previous research and this research were also found. In previous research in both patients and healthcare professionals, patients had a more positive view at eRehabilitation than the healthcare professionals [20]. It this study, that difference was not noticed, but not explored in detail as well. A possible reason for this is that healthcare professionals involved in the study of Tyagi [20] were previously involved in eRehabilitation, which was not the case for most healthcare professionals in this study. Another difference was that in our study, patient characteristics (mostly as a consequence of stroke) were reported as possible barrier, in contrast to literature about uptake of eRehabilitation not specified to stroke [33, 36]. Therefore, it is recommended that implementation strategies must be tailored to both end-users and to specific impairments of the disease as well. In order to implement interventions with the right content and sufficient ease of use, involvement of patients/informal caregivers and healthcare professionals in the development of eRehabilitation is important.

A limitation of this study is that we could not aim for data saturation among healthcare professionals. Whereas six focus groups were conducted with patients/informal caregivers and data saturation was reached, for the healthcare professionals this was not possible due to practical issues. Differences in results between patients/informal caregivers and healthcare professionals may have resulted from this imbalance. A second limitation was that the participant of the pilot study did not meet the inclusion criterion of start of rehabilitation after June 2011. This inclusion criterion was set because we believe that patients with a longer time since start of rehabilitation are not familiar with the recent stroke rehabilitation and innovations like eRehabilitation, which is a prerequisite to be able to contribute to the conversation during the focus groups. However, since the participants of the pilot study are discussing innovations in stroke rehabilitation on a regular basis, they still have a good feeling with recent developments and current stroke care. Therefore, we believe that a longer time since start of rehabilitation of these participants did not affect their opinions and statements.

Based on this study, it was not possible to determine which factors have the largest impact on the uptake of eRehabilitation, or how these are associated with characteristics of patients and healthcare professionals. Such insights are crucial since it is practically impossible to tailor an implementation strategy to all factors that may influence the uptake. To increase the uptake of eRehabilitation programs, future research should focus on such insights and factors identified as most important should be considered in the development and implementation strategy of eRehabilitation innovations for stroke rehabilitation. Those interventions should be assessed on its (cost)-effectiveness in randomized and controlled trails.

For clinical practice, we recommend that implementation strategies for eRehabilitation must be tailored to factors influencing the uptake of eRehabilitation among end-users. As a consequence of differences in the factors found between end-users, the used strategies must be different for patients/informal caregivers and healthcare professionals. For patients, this means that it is important that future eRehabilitation programs increase the ease of use, especially for of impaired body functions, to ensure eRehabilitation is applicable for as many patients as possible [37]. For uptake among healthcare professionals, it seems crucial that the eRehabilitation program is attractive, but also fits well into existing process of care. Since the uptake of eRehabilitation starts with the healthcare professional using eRehabilitation and introducing it to the patients [18], the factors mentioned by healthcare professionals should be an important starting point in increasing of uptake of eRehabilitation for, e.g., policy makers. To make sure that eRehabilitation programs have the right content and sufficient ease of use, involvement of all end-users in the development of the eRehabilitation innovation is important.

## Conclusion

This research identified factors influencing uptake of eRehabilitation in a western country. Although there was a considerable overlap in reported factors between patients/informal caregivers and healthcare professionals when it concerns eRehabilitation as innovation, this research shows that patients/informal caregivers give more emphasis to factors related to the individual patient, whereas healthcare professionals emphasize the importance of factors related to the organizational context. This difference should be considered when developing an implementation strategy.

## Abbreviations

ICT: Information and communication technology
